# Transcriptome analyses of Atlantic salmon muscle genes induced by a DNA vaccine against salmonid alphavirus, the causative agent of salmon pancreas disease (PD)

**DOI:** 10.1371/journal.pone.0204924

**Published:** 2018-10-01

**Authors:** Mehrdad Sobhkhez, Aleksei Krasnov, Børre Robertsen

**Affiliations:** 1 Norwegian College of Fishery Science, UiT The Arctic University of Norway, Tromsø, Norway; 2 Nofima AS, Norwegian Institute of Food, Fisheries & Aquaculture Research, Ås, Norway; National Cheng Kung University, TAIWAN

## Abstract

Salmonid alphavirus (SAV) is the causative agent of pancreas disease (PD) in farmed Atlantic salmon. A previous study showed that vaccination of pre-smolt salmon with a plasmid encoding the structural polypeptide of SAV gave protection against infection and development of PD accompanied by production of antibodies against the virus. In the present work we analyzed transcript responses in the muscle to vaccination with this plasmid (here named pSAV). The purpose was to shed light on how pSAV might initiate adaptive immune responses in the fish. The work was based on microarray and reverse transcription quantitative PCR analyses of muscle at the injection site 7 days after vaccination. The results showed that pSAV and pcDNA3.3 had similar abilities to up-regulate type I IFN stimulated genes. In contrast, pSAV caused higher up-regulation of IFNγ and several IFNγ inducible genes. Compared to pcDNA3.3, pSAV also gave larger increase in transcripts of marker genes for B-cells, T-cells and antigen presenting cells (APCs), which suggest attraction and role of these cells in the adaptive immune responses elicited by pSAV. Moreover, pSAV caused a stronger up-regulation of the chemokine CXCL10 and the proinflammatory cytokines IL-1ß and TNFα, which may explain attraction of lymphocytes and APCs. The present work shows that the expression profile of genes resulting from vaccination with pSAV is different from the expression profiles obtained previously by vaccination of salmonids with DNA vaccines against infectious salmon anemia virus and infectious hematopoietic necrosis virus.

## Introduction

Virus diseases cause major problems in fish farming due to economic losses and suffering of infected fish. Traditional vaccines based on inactivated virus have so far been unable to provide sufficient protection against virus diseases of fish. DNA vaccination against virus has received high expectations due to the high level of protection obtained with the DNA vaccine against infectious hematopoietic necrosis virus (IHN) in salmonids [1–3]. More recently, a DNA vaccine against infectious salmon anemia virus (ISAV) has shown a high level of protection when injected together with a plasmid encoding type I IFN from Atlantic salmon [4]. The vaccine was a plasmid expressing the virus surface protein hemagglutininesterase (HE) and the IFN plasmid was shown to function as an adjuvant. A transcriptome analysis of genes induced at the muscle injection site by a plasmid encoding HE, plasmid vector without insert, and plasmid encoding IFNa was recently carried out [5]. The work showed that plasmid DNA induced a similar set of genes as the IFNa plasmid, but at a lower level. In contrast, the HE-plasmid induced lower levels of IFN-induced genes compared to plasmid without insert. This suggested that HE expressed by the plasmid, inhibited expression of IFN-induced genes thus explaining the need for IFNa plasmid as adjuvant. Salmonid alphavirus (SAV) is the causative agent of pancreas disease (PD) in farmed Atlantic salmon. A DNA vaccine against SAV was recently shown to give significantly higher protection of Atlantic salmon against pancreas disease (PD) than a commercial vaccine based on inactivated virus [6]. This vaccine is a plasmid encoding the structural polyprotein C-E3-E2-6K-E2 of SAV3 (here named pSAV), which upon uptake in cells results in expression of E2 on the surface of the cells. In contrast to the ISAV DNA vaccine, the SAV3 DNA vaccine did not need addition of IFN plasmid to achieve protection. In the present study we did transcriptome analyses of muscle at the injection site of pSAV, pcDNA3.3 and PBS 7 days after injection. This was performed by microarray analysis and reverse transcription quantitative PCR (RT-qPCR).

The purpose of the study was to find out whether expression of the structural proteins of SAV3 have unique effects on gene transcription that might shed light on how this DNA vaccine influences the immune system. It is likely that the adaptive immune system reacts to antigens as soon as they are expressed. Day 7 post injection was thus chosen as time point for harvest of mRNA since it represents early high level expression of plasmid encoded genes in the muscle [5, 7, 8].

The data showed that vaccination with pSAV and the vector plasmid pcDNA3.3 gave similar up-regulation of type I IFN stimulated genes (ISGs), which is different from the HE-plasmid of SAV. In contrast, pSAV caused higher up-regulation of IFNγ and several IFNγ inducible genes compared to pcDNA3.3. Moreover, pSAV gave increase in transcripts of B- cells, T-cells and antigen-presenting cells (APCs) presumably due to attraction of these cells by up-regulation of inflammatory chemokines and cytokines.

## Materials and methods

### Ethics statement

All fish experiments were approved by the Norwegian Animal Research Authority (NARA) and Norwegian “Regulation on Animal Experimentation” was observed during fish handling.

### Fish

Atlantic salmon (*Salmo salar* L.) presmolts (30–40 g), Aquagen standard (Aquagen, Kyrksæterøra, Norway), were kept at 10°C in 300 L tanks supplied with fresh water and were fed commercial dry food in Tromsø Aquaculture Research Station, Norway. For vaccination, fish were anesthetized with 0.005% benzocaine (ACD Pharmaceuticals, Norway). Different groups of fish were labeled by tattooing (2% alcian blue, Panjet inoculator). For harvest of muscle samples, fish were euthanized by an overdose of benzocaine (0.01%).

### Treatment of fish with plasmids

pcDNA3.3 encoding the open reading frame (ORF) for SAV3 complete structural polyprotein C‐E3‐E2‐6K‐E1 (pSAV) is based on the genome sequence of the SAV3 H10 isolate (GenBank Accession number: JQ799139) and has previously been named pCSP [6]. pcDNA3.3 without insert was used as control. The plasmids were purified by EndoFree plasmid purification kit from Qiagen. Presmolt salmon were injected intramuscularly (i.m.) approximately 1 cm below the dorsal fin with 15 μg of plasmid in 50 μl sterile PBS pH 7.4 or with 50 μl PBS only. Fish groups (N = 5) were injected with PBS (Group 1), pcDNA3.3 (Group 2) and pSAV (Group 3). Muscle samples from the injection site were harvested one week post injection and used for RNA isolation.

### Reverse transcription quantitative PCR (RT-qPCR)

Muscle samples from the injection site were homogenized using TissueLyser II (QIAGEN) (30 sec, 30 1/s) and total RNA was extracted using the RNeasy® Mini Kit (Qiagen). cDNA was produced using high capacity cDNA Reverse Transcription kit (Applied Biosystems) (500 ng RNA in a 20 μl reaction). cDNA was diluted 10 times and 5 μl of cDNA was used per 20 μl PCR reaction, which also included primers for target gene (0.25 μM) and Fast SYBR® Green Master Mix (Applied Biosystems). The samples were applied in duplicates and the measurement was done using ABI Prism 7500 FAST Cycler from Applied Biosystems (initial denaturation 95°C: 20s and 40 cycles of 95°C: 3s, and 60°C: 30s). Primer sequences are included in S1 Table. Expression values were normalized against the levels of EF1αβ and fold change in expression of different genes was calculated against that of PBS group using the method described by Pfaffl [9]. Unpaired t-test with two-tail distribution was used for statistical analysis, *p* ≤ 0.05.

### Microarray analysis

The analyses were carried out essentially as described earlier [5]. Nofima’s Atlantic salmon oligonucleotide microarray SIQ-6 (GEO Accession no. GPL16555) was produced by Agilent Technologies in the 15 K x 8 format, all reagents and equipment were from the same source [10]. Analyses included three groups, four individuals per group at one time-point; totally twelve arrays were used. RNA was isolated as described for RT-qPCR. Total RNA (200 ng per reaction) was labelled with Cy3 using Low Input Quick Amp Labeling Kit and fragmented with Gene Expression Hybridization Kit. Hybridization was performed for 17 hours in an oven at 65°C at rotation speed of 10 rounds per minute. Arrays were washed for one minute with Gene Expression Wash Buffer I at room temperature, and one minute with Gene Expression Wash Buffer II at 37°C and scanned. Data analyses were carried out with Nofima’s bioinformatics package [10]. Global Normalization was performed by equalizing the mean intensities of all microarrays. Next, the individual values for each feature were divided to the mean value of all samples producing expression ratios (ER). The log_2_-ER were calculated and normalized with the locally weighted non-linear regression (Lowess). The data are presented as ratios of plasmid injected groups to PBS injected control. Differentially expressed genes were selected by criteria: p < 0.05 and log_2_-ER > |0.8| (1.74-fold). The microarray data presented in this publication has been deposited in the NCBI´s Gene Expression Omnibus (GEO, https://www.ncbi.nlm.nih.gov/geo/) and is available under the accession number GSE115689.

## Results

### IFN response

**Fig 1:**
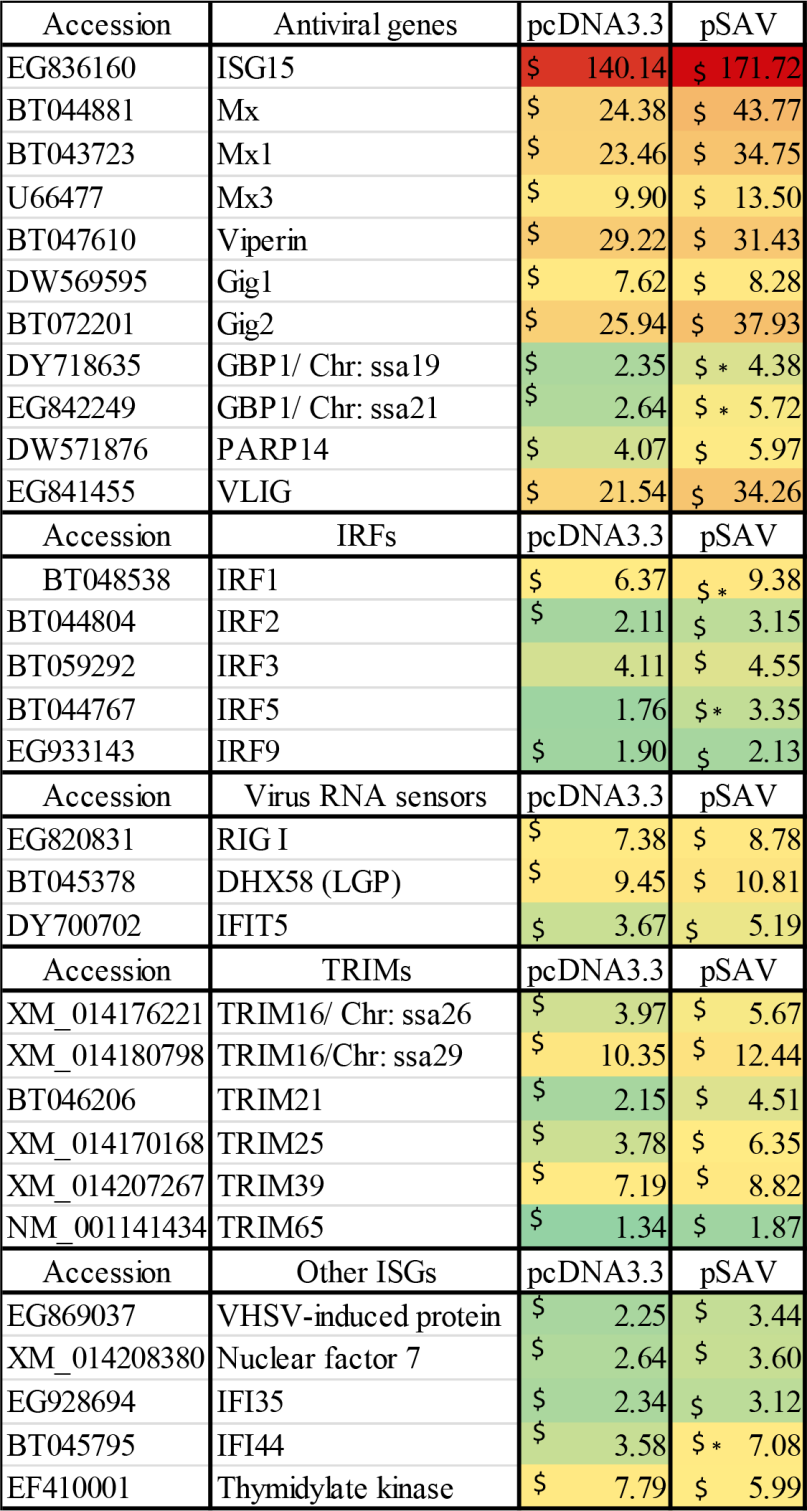
Expression of IFN stimulated genes (ISGs) in muscle in response to pcDNA3.3 and pSAV measured by microarray analysis. The ISGs include the main antiviral genes, interferon regulatory factors (IRFs), virus RNA sensors and tripartite motif-containing proteins (TRIMS). Groups of fish (N = 4) were injected with PBS, pcDNA3.3 and pSAV. RNA was isolated from muscle at the injection site 7 days post injection. Numbers represent fold changes compared to the PBS group. Symbols $ and * denote significant difference (p ≤ 0.05) between PBS and plasmid groups, and between pcDNA3.3 and pSAV groups, respectively.

**Fig 2:**
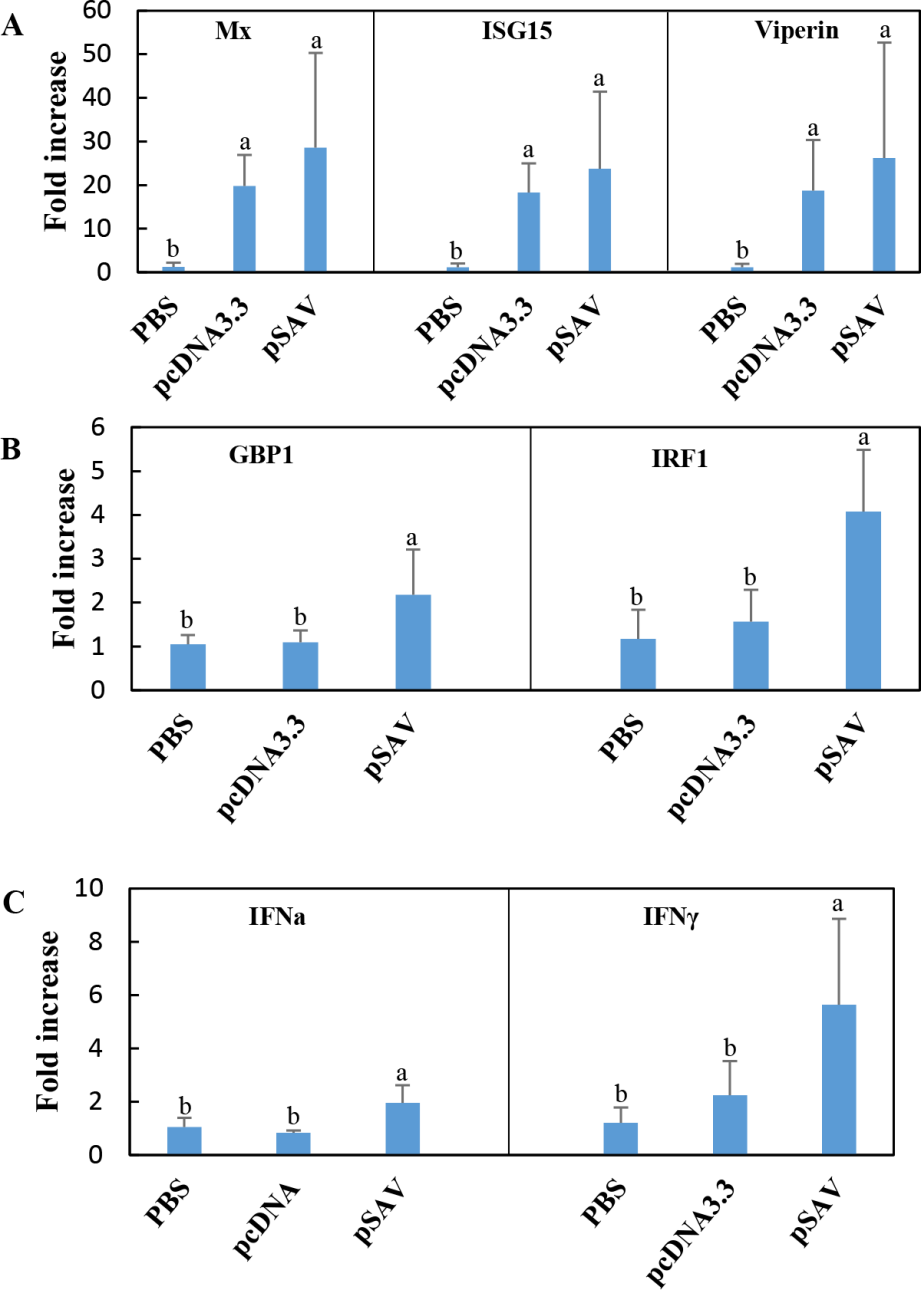
**Expression of type I IFN inducible genes (A), IFNγ inducible genes (B), and type I IFN (IFNa) and type II IFN (IFNγ) genes (C) in response to vaccination with plasmids.** Groups of fish (N = 5) were injected with PBS, pcDNA3.3 and pSAV. RNA was isolated from muscle at the injection site 7 days post injection and expression of different IFNs and ISGs was measured by RT-qPCR. Expression value of each gene was normalized against the levels of EF1αβ. Numbers represents fold increase in expression (mean ± SD) compared to the PBS group. Bars not sharing common letter indicate significant difference (p ≤ 0.05).

Because type I IFNs (IFN-I) enhance adaptive immune responses and function as adjuvants in the DNA vaccine against ISAV [4], we wanted to study whether pSAV had any effect on IFN-I induced genes (ISGs) during DNA vaccination. The microarray data showed that pcDNA3.3 and pSAV had similar ability to up-regulate typical ISGs such as ISG15, Mx and viperin (Fig 1), which was confirmed by RT-qPCR (Fig 2A). A similar pattern of response was seen for a several other ISGs (Fig 1). In contrast pSAV compared to pcDNA3.3 gave significantly higher up-regulation of guanylate binding protein 1 (GBP1), interferon regulatory factor 1 (IRF1), IRF5 and IFN- inducible protein IFI44 (Fig 1). This was confirmed by RT-qPCR for GBP1 and IRF1 (Fig 2B). GBP-1 is an antiviral and antibacterial GTP-ase while IRF1 activates transcription of IFN-I and viperin [11–14]. IRF5 is involved in induction of IFN-I, chemokines and proinflammatory cytokines [15–17]. IFI44 has anti-proliferative functions [18]. Notably, GBP1 and IRF1 are more strongly induced by IFNγ (type II IFN) than by IFN-I in both mammals and Atlantic salmon [19, 20]. Indeed, RT-qPCR showed that pSAV up-regulated IFNγ more strongly than pcDNA3.3 (Fig 2C), which supports that GBP1 and IRF1 were induced by IFNγ in the pSAV group. RT-qPCR showed that the IFN-I IFNa was slightly up-regulated by pSAV, but not by pcDNA3.3 (Fig 2C).

### Attraction of B- and T-cells

**Fig 3:**
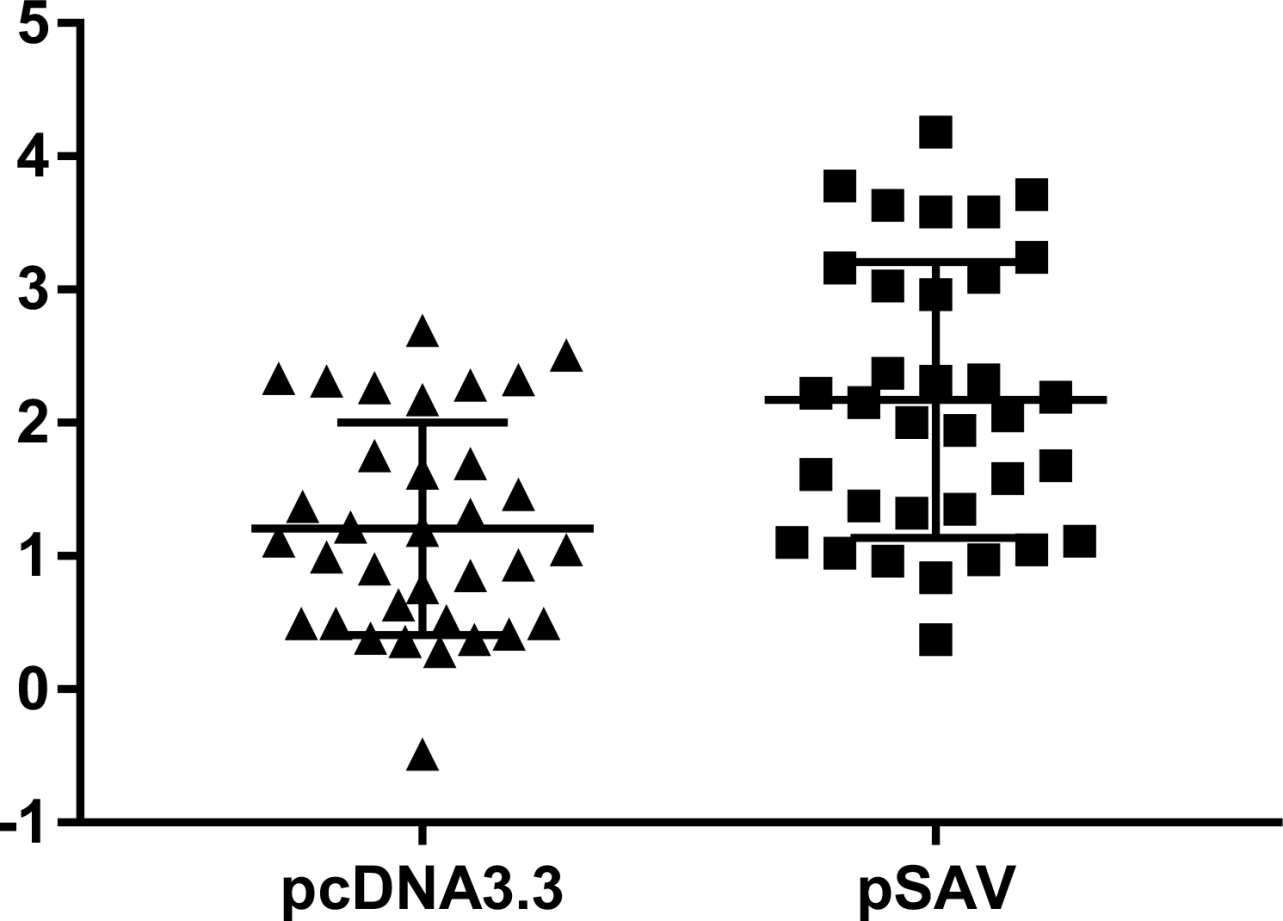
B cell specific genes, microarray results. Markers correspond to probes representing constant regions of IgM, IgT and IgD and segments of light and heavy chains (totally 34 probes). Data are log2-ER (expression ratio) to PBS control. Difference between the groups is significant (p<0.0001, paired t test).

**Fig 4:**
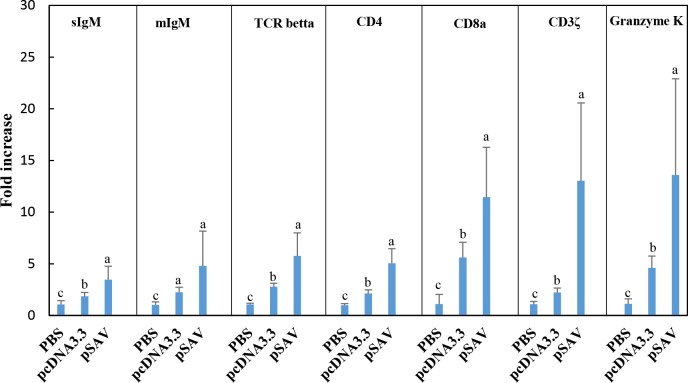
Expression of T- and B- cell marker genes in response to injection of plasmids measured by RT-qPCR. Treatment groups and experimental details as described in Fig 2. Expression value of each gene was normalized against the levels of EF1αβ. Numbers represents fold increase in expression (mean ± SD) compared to the PBS group. Bars not sharing common letter indicate significant difference (p ≤ 0.05).

Protection of salmon against SAV by vaccination with pSAV, could be due to both increased antibody response and increased production of specific cytotoxic T cells. We thus wanted to find out if pSAV vaccination was associated with increased attraction of B- and T-cells to the muscle injection site, which should be revealed by gene markers for these cells. The microarray data showed that both pcDNA3.3 and pSAV enhanced transcripts encoding constant regions of IgD, IgM and IgT and segments of the heavy and light chains, totally 34 probes (Fig 3), the increase in the pSAV group was 1.95-fold greater compared to the pcDNA3.3 group. RT-qPCR analysis of membrane bound IgM (mIgM) and soluble IgM (sIgM) confirmed up-regulation by both plasmids, but only expression of sIgM was significantly higher in the pSAV group (Fig 4).

**Fig 5:**
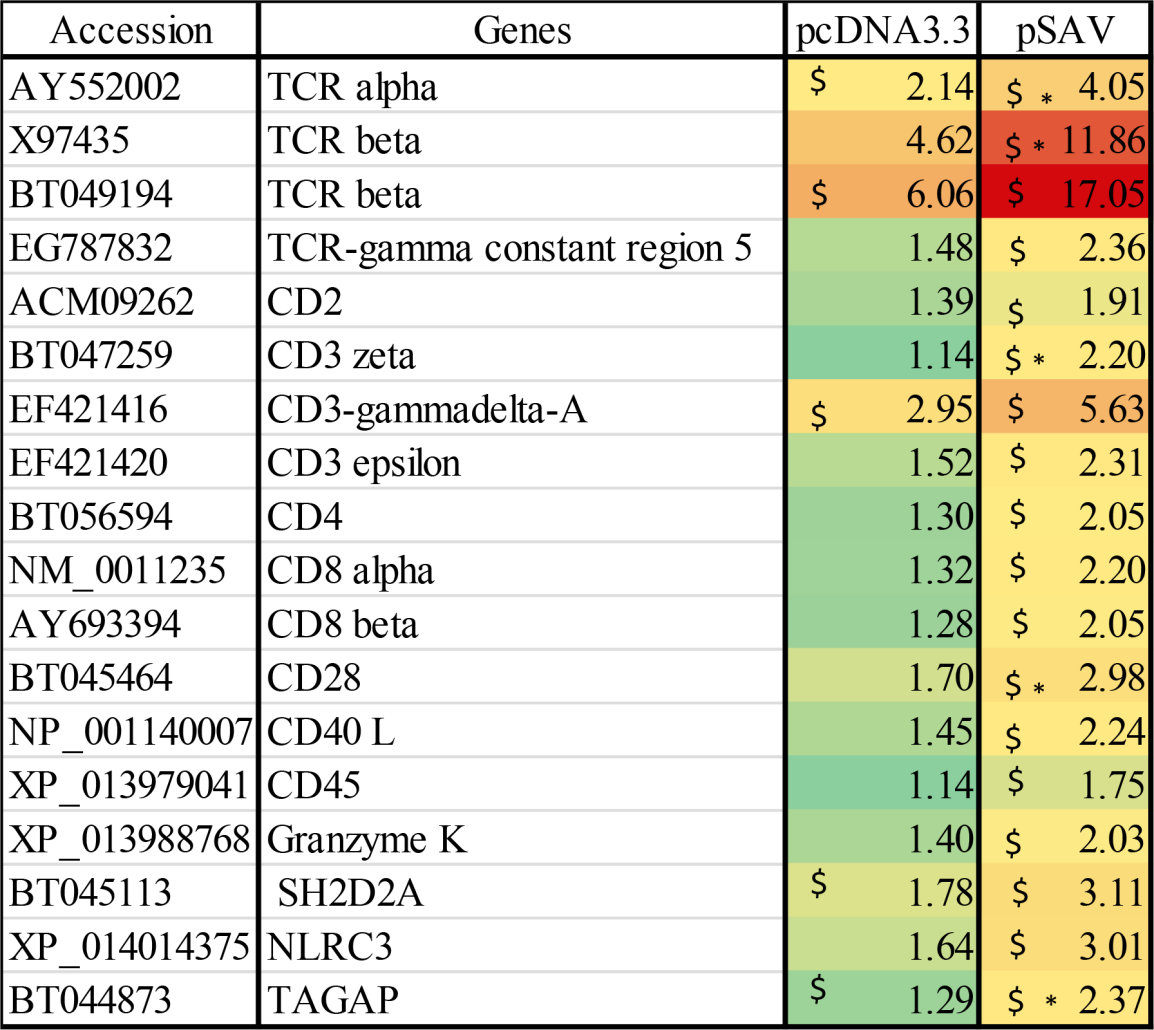
Microarray data of T cell marker genes. Data produced and presented as explained in Fig 1.

The microarray data showed that expression of T-cell receptor (TCR) alpha and beta chains were increased by both plasmids, but were significantly higher up-regulated by pSAV than by pcDNA3.3 (Fig 5). This was confirmed by RT-qPCR for TCRbeta (Fig 4). The microarray analysis also showed that compared to pcDNA3.3, pSAV caused significant higher increase in transcripts for other typical T-cell markers (Fig 5). This was confirmed by RT-qPCR for CD4, CD8a, CD3z and granzyme K (Fig 4). Taken together these data suggests that B- and T-cells are attracted to the muscle injection site by plasmid DNA and that pSAV had a stronger attractive effect than pcDNA3.3, especially on T-cells.

### MHC I antigen presentation pathway

**Fig 6:**
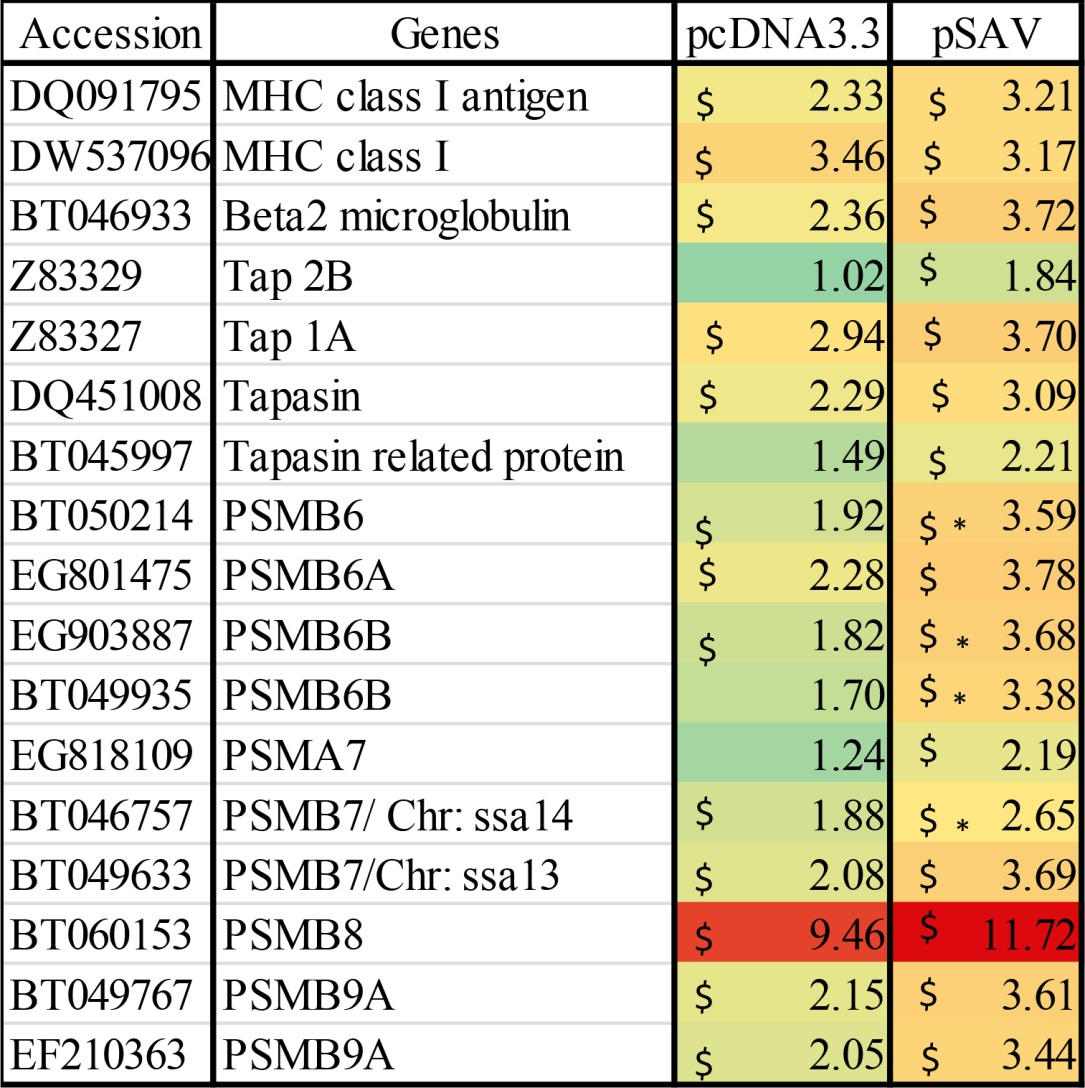
Microarray data showing expression of genes involved in MHC I antigen presentation. Data produced and presented as explained in Fig 1.

**Fig 7:**
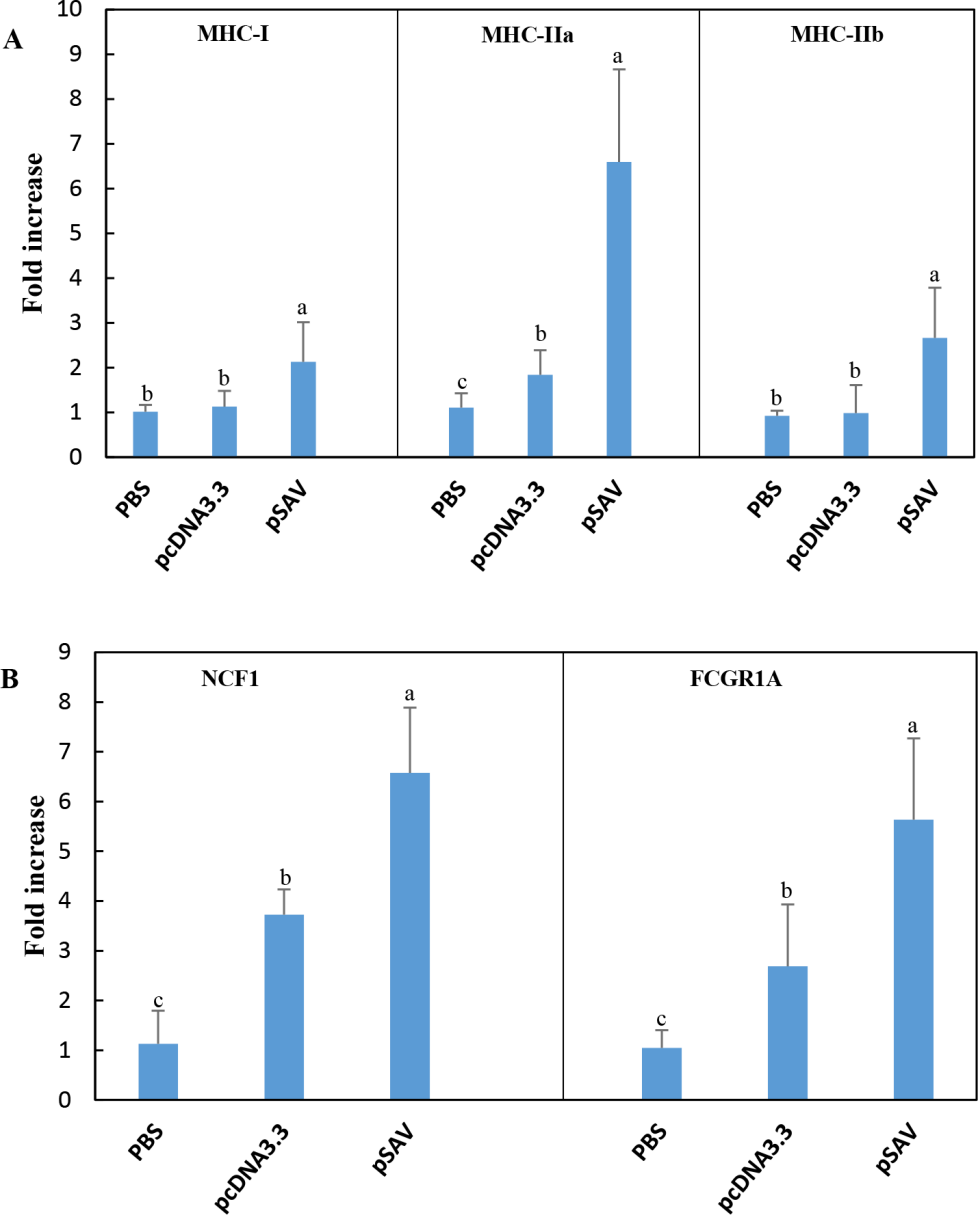
Expression of genes involved in antigen presentation / phagocytosis in response to plasmids measured by RT-qPCR. Treatment groups and sampling for RNA extraction as described in Fig 2. Expression value of each gene was normalized against the levels of EF1αβ and fold increase in expression of different genes was calculated against that of the PBS group. Bars not sharing common letter indicate significant difference (p ≤ 0.05).

Most cells have the ability to present antigen to cytotoxic T-cells through MHC I, which occurs by digestion of antigen by the proteasome, transportation of peptides into ER by TAP followed by loading of antigenic peptides onto MHC-I involving tapasin and other chaperone molecules [21]. As shown in Fig 6, the microarray data showed that pcDNA3.3 and pSAV up-regulated to a similar extent MHC I, beta-2-microglobulin and PSMB8, which is a subunit of the proteasome. This was confirmed by RT-qPCR for MHC I (Fig 7A). Other proteasome subunits (PSMB6B and PSMB7) were significantly higher up-regulated by pSAV than by pcDNA3.3 (Fig 6). Higher transcript values were also observed for TAP and tapasin in the pSAV group, but the differences between pSAV and pcDNA3.3 were not significant for these genes. Interestingly, PSMB6, PSMB7, TAP and tapasin are all IFNγ -induced genes [22–25].

### Antigen presenting cells (APCs) and the MHC II antigen presentation pathway

**Fig 8:**
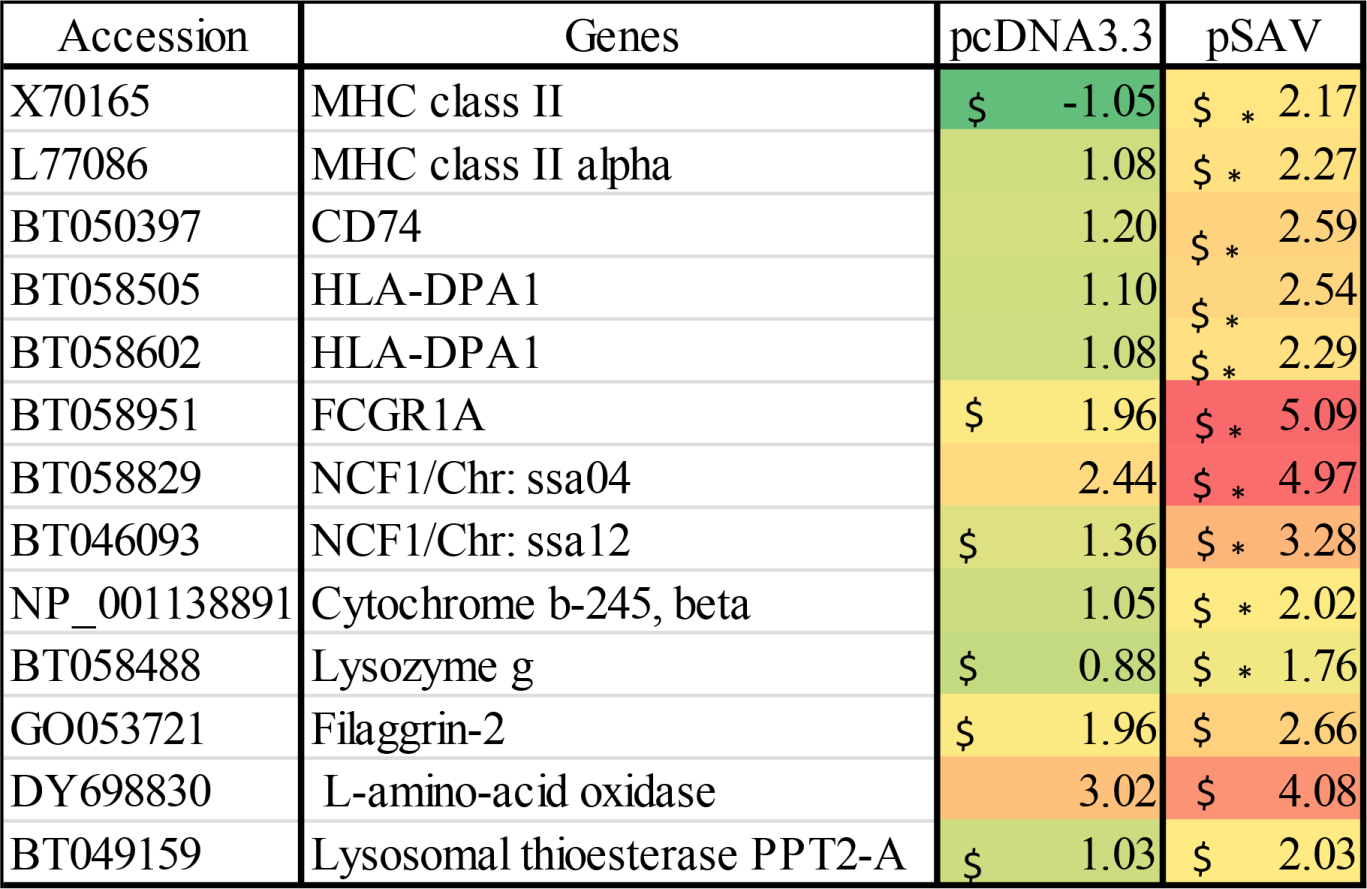
Microarray data showing expression of marker genes for antigen presenting cells (APC) / phagocytic cells. Data produced and presented as explained in Fig 1.

Since attraction of APCs might indicate that antigen presentation to T-cells occurs at the muscle injection site, we analysed expression of genes for surface markers of APCs. Extracellular protein antigens are taken up by APCs and processed by endolysosomal proteases into peptides, which are loaded onto MHC II molecules by displacing the peptide CLIP derived from the class II-associated invariant chain, which is also known as MHC II gamma chain or CD74 when it is surface expressed [26]. The microarray data showed that compared to pcDNA3.3, pSAV up-regulated more strongly MHC II alpha and beta chains and CD74 (Fig 8). RT-qPCR confirmed stronger up-regulation of MHC II by pSAV (Fig 7A). One of the most prominent genes that showed higher transcript levels with pSAV was high affinity Ig receptor I (FcγRI), which is encoded by FCGRI (Figs 7B and 8). This protein is expressed on monocytes, macrophages and dendritic cells (DCs) and can be up-regulated by IFNγ [27, 28].

### Effector molecules of APCs/phagocytic cells

pSAV showed higher transcript levels of lysozyme G, cytochrome b-245 beta chain and neutrophil cytosol factor (NCF1), also known as p47phox (Fig 8). Lysozymes are responsible for degradation of peptidoglycans in the cell wall of gram-positive and gram-negative bacteria. NCF1 is the cytoplasmic subunit of NADPH-oxidase complex in neutrophils and macrophages. NCF1 has a central role in oxidative burst, which leads to production of superoxide anion and formation of antimicrobial reactive oxygen species [29]. Cytochrome b-245 is a membrane-bound component of the NADPH-oxidase complex [30]. RT-qPCR confirmed that NCF1 was significantly higher expressed in response to pSAV compared to pcDNA3.3 and PBS (Fig 7B). Altogether the increase in transcripts of this group of genes suggests attraction of phagocytic cells like macrophages or neutrophils to the site of pSAV injection.

### Other leukocyte markers

Several other genes, which are commonly expressed by lymphocytes and other leukocytes were significantly higher up-regulated by pSAV than by pcDNA3.3 (S1 Fig). These included plastin-2, Wiskott-Aldrich syndrome protein (WASP) and Src kinase-associated phosphoprotein 2 (Skap-2). Plastin-2 is specifically expressed in hematopoietic cell lineage and has important roles in processes related to neutrophil biology and function, stabilization of immune synapse (IS) between MHCII and TCR, T-cell activity as well as T-cell and B-cell motility and macrophage activity [31]. WASP is necessary for many lymphoid immune cell functions like phagocytosis, NK-cell cytotoxicity, TCR/BCR downstream signaling and monocyte chemotaxis [32, 33]. Skap-2 is involved in macrophage cytoskeletal rearrangement [34].

### Chemokines and pro-inflammatory cytokines

**Fig 9:**
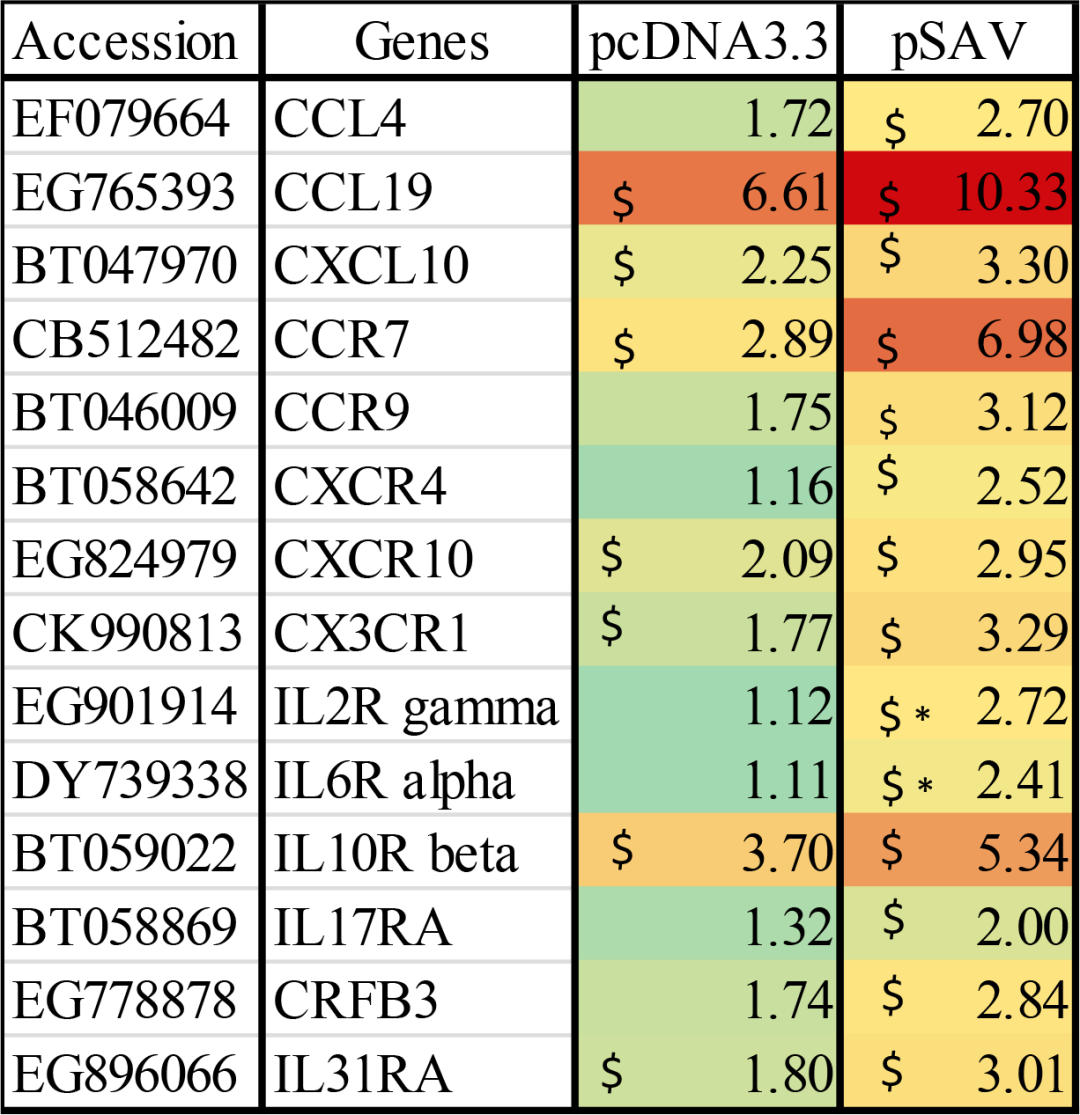
Microarray data of chemokines, cytokines and their receptors. Data produced and presented as for Fig 1.

**Fig 10:**
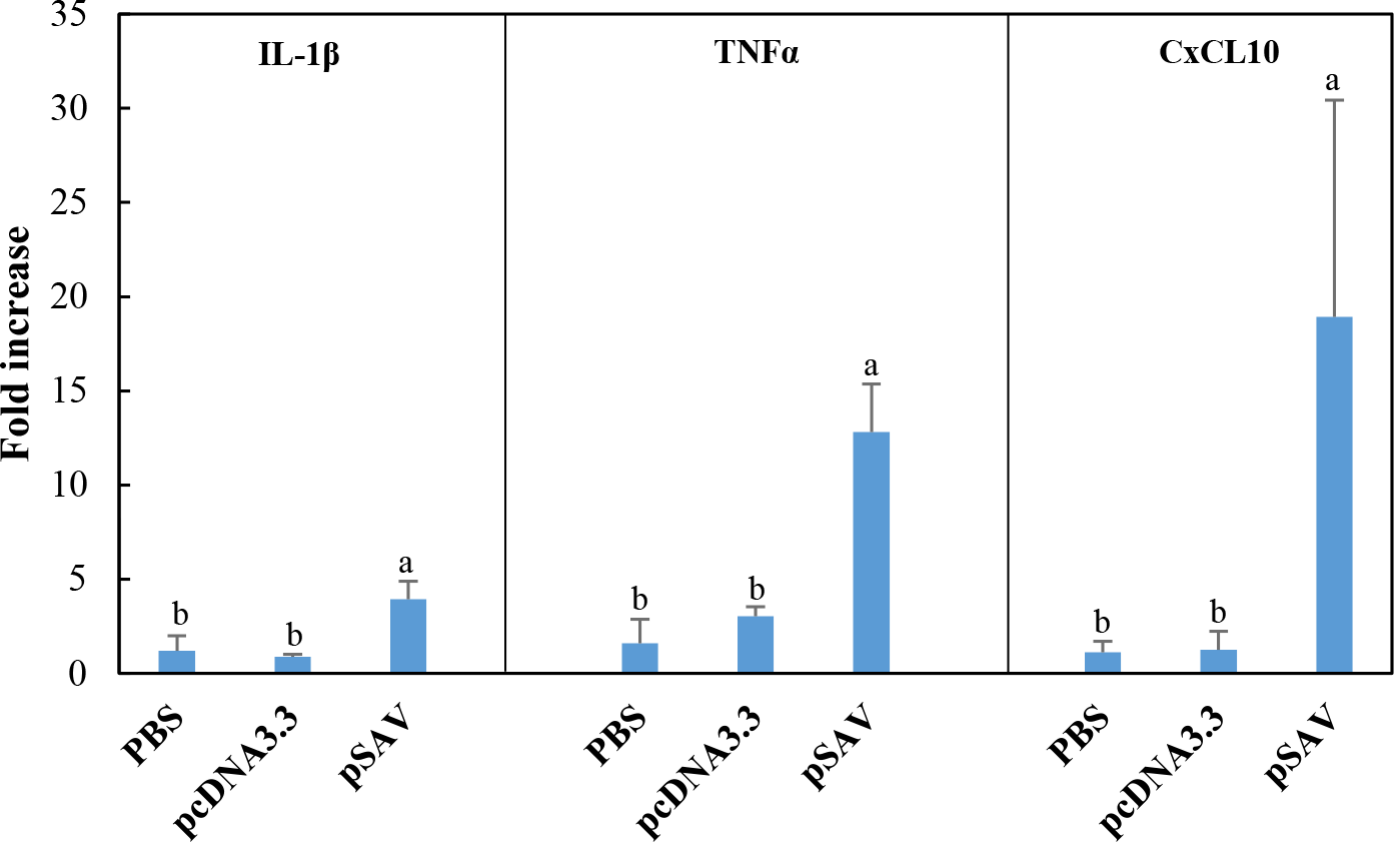
Expression of chemokines and inflammatory cytokines in response to plasmids measured by RT-qPCR. Treatment groups and sampling for RNA extraction as described in Fig 2. Expression value of each gene was normalized against the levels of EF1αβ and fold change in expression of different genes was calculated against that of the PBS group. Bars not sharing common letter indicate significant difference (p ≤ 0.05).

Attraction of leukocytes to the plasmid injection site must be mediated by chemokines and proinflammatory cytokines [35]. Microarray data for chemokines, chemokine receptors and cytokine receptors that showed up-regulation with pcDNA3.3 and pSAV are presented in Fig 9. The data showed higher transcript values for the chemokines CCL4, CCL19 and CXCL10 in response to pSAV compared to pcDNA3.3, but the differences were not significant. In general, cytokines showed low upregulation in response to plasmids in the microarray analysis. RT-qPCR analyses showed that CXCL10 and the proinflammatory cytokines IL-1ß and TNFα were more strongly up-regulated by pSAV than by pcDNA3.3 (Fig 10). Up-regulation of CXCL10 may be mediated by IFNγ as suggested by previous work [19]. Of cytokine receptors, the microarray data showed higher up-regulation of IL2R and IL6R by pSAV than by pcDNA3.3.

## Discussion

A previous study showed that i.m. injection of Atlantic salmon with a plasmid expressing the whole SAV3 structural polyprotein (here named pSAV) gave strong protection against development of pancreas disease caused by SAV3 infection accompanied by production of IgM antibodies against the virus [6]. The present study was conducted to find out if transcriptome analysis of muscle at the injection site might reveal a pattern of gene expression that could shed light on the protective effect of the vaccine. DNA vaccination against a virus results in uptake of plasmid and expression of virus protein both by muscle cells and resident APCs [36]. Changes in immune gene expression at the muscle injection site might be due to innate responses to plasmid DNA and to both innate and adaptive immune responses to the expressed viral proteins. The transcriptome data in this work demonstrated that pSAV and pcDNA3.3 had similar abilities to up-regulate typical IFN-I inducible genes (ISGs). The IFN-I response to pSAV is thus likely to be a response to plasmid DNA rather than to the viral proteins. The virus proteins expressed by pSAV do thus not seem to induce IFN-I such as the G-protein of the DNA vaccine against IHNV [5, 8]. Moreover, pSAV does not inhibit the IFN-I response unlike hemagglutininesterase in the DNA vaccine against ISAV [5, 8]. In fact, pSAV induced a distinct set of genes in the muscle compared to the other two DNA vaccines. Compared to pcDNA3.3, pSAV firstly caused higher up-regulation of IFNγ, which was supported by higher up-regulation of several IFNγ inducible genes. Secondly, pSAV caused larger expression of marker genes for T-cells, B-cells and APCs, which might all be important for development of the adaptive immune responses against the SAV3. Thirdly, pSAV caused a stronger up-regulation of the chemokine CXCL10 and proinflammatory cytokines IL-1ß and TNFα which may explain attraction of lymphocytes and APCs.

### IFNγ response

One of the most prominent responses to pSAV was increased expression of IFNγ (Fig 2C) and the IFNγ inducible genes IRF-1, GBP-1 and CXCL-10 (Figs 1, 2B and 9). This suggests that expression of the SAV3 structural proteins results in increased IFNγ production at the muscle injection site. IFNγ is regarded as a typical Th1 cytokine because it directs differentiation of naïve CD4^**+**^ cells toward a Th1 phenotype and is a major product of CD4^**+**^ T helper 1 (Th1) cells and CD8^**+**^ cytotoxic T cells [20]. Whether Th1 or cytotoxic T cells are the source of IFNγ is, however, uncertain since 7 days post vaccination is very early in the development of an adaptive immune response in cold water fish. More likely sources of IFNγ at this stage are natural killer (NK) cells, APCs and natural killer T (NKT) cells [20, 37]. Little is yet known about NK and NKT cells in fish, however. Whichever source, enhanced production of IFNγ might increase antigen presentation as discussed below, and thus influence the differentiation of T-cells at the injection site.

### Attraction of B- and T-cells

The data for pcDNA3.3 suggests that B-cells and T-cells are attracted to the plasmid injection site by recognition of plasmid DNA by muscle cells as described in previous work [4]. Higher mean transcript values for B-cell markers (IgM, IgD and IgT) were observed for the pSAV group compared to pcDNA3.3, but the differences for the individual genes were not significant. On the other hand, both the microarray and RT-qPCR analysis suggest increased attraction of T-cells to the pSAV injection site compared to pcDNA3.3, due to significantly increased transcript levels of TCR chains, CD4, CD8 and CD3zeta. Attraction of cytotoxic T-cells by pSAV is suggested by increased expression of CD8a and granzyme K. Taken together, it is interesting to note that the T-cell response to pSAV appears to be more prominent than the B-cell response. Whether cytotoxic T-cells are involved in the protective immune response to SAV infection should thus be investigated in future studies.

### APCs and genes involved in antigen presentation

Professional APCs include dendritic cells, macrophages and B-cells [26]. Participation of APCs in initiation of the adaptive immune response against SAV3 structural proteins is supported by the fact that compared to pcDNA3.3, pSAV increased more strongly expression of MHC II, CD74 and FcγRI (Figs 7 and 8). These molecules are constitutively expressed in professional APCs, but may be up-regulated by IFNγ in professional and non-professional APCs [20, 38]. pSAV may thus have a positive effect on antigen presentation through the MHC II pathway.

In addition, pSAV gave higher transcript values for several genes that are involved in the MHC I antigen presentation pathway (PSMB6, PSMB7, LMP-7, TAP1A, TAP2B and tapasin) and which may be induced by IFNγ [20]. This suggests a more efficient presentation of virus antigens through the MHC-I pathway possibly leading to cytotoxic T-cell production against the virus. Notably, pSAV and pcDNA3.3 showed similar increases in MHC I heavy chain, which suggests that MHC I expression is mainly triggered by plasmid DNA. MHC I is expressed by most cells in mammals, but can be up-regulated by IFN-I and IFNγ [39, 40]. Taken together, pSAV appears to attract APCs and/or induce genes in resident APCs, which provide a more efficient presentation of virus antigen to T-cells through both MHC pathways.

### Chemokines and pro-inflammatory cytokines

Attraction of leukocytes to the plasmid injection site must be mediated by chemokines and/or proinflammatory cytokines [35]. The RT-qPCR analyses suggest that CXCL10, IL-1ß and TNFα, are likely to be involved in attraction of T-cells, B-cells and APCs since they are more strongly up-regulated by pSAV than by pcDNA3.3. Up-regulation of CXCL10 may be mediated by IFNγ as suggested by previous work [19]. IL-1ß and TNFα are key pro-inflammatory cytokines [35, 41].

## Conclusion

While previous work showed that vaccination of Atlantic salmon with pSAV leads to production of antibodies against SAV, the present study suggests that pSAV vaccination also results in a strong T-cell response due to attraction of T-cells, APCs and up-regulation of IFNγ. Whether the antibody response to the SAV3 surface protein E2 is T-dependent or T-cell independent or whether cytotoxic T-cells are involved in protection against SAV3 is not known. This study thus motivates future studies of cytotoxic T-cell responses to the DNA vaccine against SAV.

The data support the hypothesis that the muscle injection site is a meeting place for B-cells, T-cells and APCs that is important for initiation of the adaptive immune response by the DNA vaccine against SAV3. In mammals, activation of B- and T-cells might occur at the muscle injection site, but plasmid transfected APCs also travel to lymph nodes for expression of antigen and activation of B- and T -cells [36]. While fish do not possess lymph nodes, it cannot be excluded that plasmid-transfected APCs travel to the head kidney and/or spleen for activation of lymphocytes.

With these data, it can be concluded that the DNA vaccines against the viruses IHNV, ISAV and SAV, each have unique transcript profiles at the muscle injection site 7 days after vaccination. The G-protein vaccine against IHNV shows a strong IFN-I stimulatory profile while the ISAV HE vaccine shows a profile of IFN-I inhibition [5, 8]. In contrast, the SAV3 vaccine shows neither positive nor negative effects on IFN-I induced genes while it shows a clear IFNγ stimulatory profile.

## Supporting information

S1 Fig Microarray data showing expression of various leukocyte marker genes. Data produced and presented as explained in Fig 1.
(TIF)

S1 Table a. List of primers used for RT-qPCR with SYBR Green. b. List of primers used for RT-qPCR with TaqMan.
(DOCX)
