# Supplementary figures and images for: Transcriptome analyses of Atlantic salmon muscle genes induced by a DNA vaccine against salmonid alphavirus, the causative agent of salmon pancreas disease (PD)
Source: PLoS One. 2018 Oct 1;13(10):e0204924. doi: 10.1371/journal.pone.0204924 (PMC6166962; doi:10.1371/journal.pone.0204924)

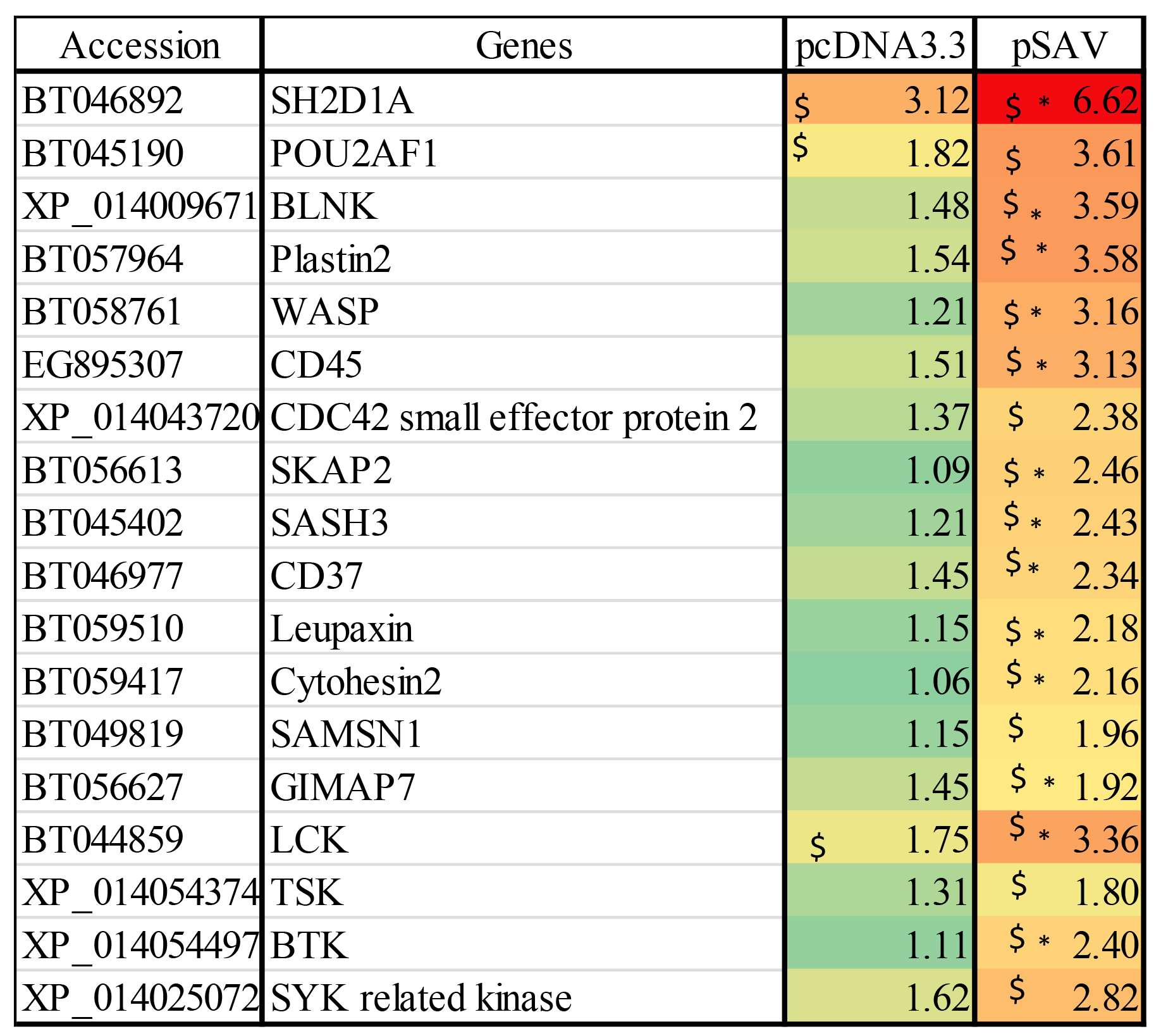

Supplement: S1 Fig — Data produced and presented as explained in Fig 1. (TIF) [file pone.0204924.s001.tif]
