# Supplementary material for: Transcriptome analyses of Atlantic salmon muscle genes induced by a DNA vaccine against salmonid alphavirus, the causative agent of salmon pancreas disease (PD)
Source: PLoS One. 2018 Oct 1;13(10):e0204924. doi: 10.1371/journal.pone.0204924 (PMC6166962; doi:10.1371/journal.pone.0204924)
Supplement: S1 Table — a. List of primers used for RT-qPCR with SYBR Green. b. List of primers used for RT-qPCR with TaqMan. (DOCX) [file pone.0204924.s002.docx]

Table S1

a. List of primers used for RT-qPCR with SYBR Green

| Gene | Sequence | Accession |
| --- | --- | --- |
| EF1a Fw | TGCCCCTCCAGGATGTCTAC | BG933853 |
| EF1a Rev | CACGGCCCACAGGTACTG |  |
| IFNa1 Fw | TGCAGTATGCAGAGCGTGTG | DQ354152 |
| IFNa1 Rev | TCTCCTCCCATCTGGTCCAG |  |
| IFNγ Fw | AAGGGCTGTGATGTGTTTCTG | AY795563 |
| IFNγ Rev | TGTACTGAGCGGCATTACT |  |
| Mx1 Fw | TGCAACCACAGAGGCTTTGAA | U66475 |
| Mx1 Rev | GGCTTGGTCAGGATGCCTAAT |  |
| ISG15 Fw | CTGAAAAACGAAAAGGGCCA | AY926456 |
| ISG15 Rev | GCAGGGACTCCCTCCTTGTT |  |
| Viperin Fw | TCCTTGATGTTGGCGTGGAA | BT047610 |
| Viperin Rev | GCATGTCAGCTTTGCTCCACA |  |
| IRF1 Fw | GCAATGAAGTAGGCACAGCA | BT048538 |
| IRF1 Rev | CGCAGCTCTATTTCCGTTTC |  |
| GBP1 Fw | GGTGGTCGGGCTGTACCGC | BT059640 |
| GBP1 Rev | CAGGGTGAGGCACACACCAC |  |
| CD4 Fw | GTTGAAAGGGCGAAAGTGAG | NM_001146408 |
| CD4 Rev | GTGCCTTCGATGAGGACATT |  |
| CD8a Fw | CGTCTACAGCTGTGCATCAATCAA | NM_001123583 |
| CD8a Rev | GGCTGTGGTCATTGGTGTAGTC |  |
| TCRβ Fw | AAGACTGGCACAACCCAGAC | X97435 |
| TCRβ Rev | CAGTCTGGGTGCTCTTCACA |  |
| CD3z Fw | ATTCTGGATGGCTTCCTCCT | NM_001123620 |
| CD3z Rev | TATTCGCCCATAACCACCTC |  |
| Granzyme K Fw | CCAGGACATGCTCTGTGCTA | XM_014133293 |
| Granzyme K Rev | CTTTTGGAGAACCGGGTGTA |  |
| MHCI Fw | GAAGAGCACTCTGATGAGGACAG | JN561338 |
| MHCI Rev | CACCATGACTCCACTGGGGT |  |
| MHCIIa Fw | TCTCCAGTCTGCCCTTCACC | BT150000 |
| MHCIIa Rev | GAACACAGCAGGACCCACAC |  |
| MHCIIb Fw | ATGGTGGAGCACATCAGCC | ABX44766 |
| MHCIIb Rev | CTCAGCCTCAGGCAGGGAC |  |
| FCGR1 Fw | GGGTGGAGCTTCAGAGACAG | BT058649 |
| FCGR1 Rev | CATGGATCAGACGGTCATTG |  |
| IL1β Fw | GCTGGAGAGTGCTGTGGAAGA | XM_014170479 |
| IL1β Rev | TGCTTCCCTCCTGCTCGTAG |  |
| CxCL10 Fw | TGAAGAACGGAAAAGGGATG | EF619047 |
| CxCL10 Rev | TCAGGGCACTGACTCAACTG |  |
| TNFα Fw | TGCTGGCAATGCAAAAGTAG | AY848945 |
| TNFα Rev | AGCCTGGCTGTAAACGAAGA |  |

b. List of primers used for RT-qPCR with TaqMan

| Gene | Sequence | Accession |
| --- | --- | --- |
| EF1aB probe | AAATCGGCGGTATTGG | BG933853 |
| EF1aB Fw | TGCCCCTCCAGGATGTCTAC |  |
| EF1aB Rev | CACGGCCCACAGGTACTG |  |
| sIgM Fw | CTACAAGAGGGAGACCGGAG | Y12457 |
| sIgM Rev | AGGGTCACCGTATTATCACTAGTTT |  |
| sIgM probe | TCCACAGCGTCCATCTGTCTTTC |  |
| mIgM Fw | CCTACAAGAGGGAGACCGA | BT059185 |
| mIgM Rev | GATGAAGGTGAAGGCTGTTTT |  |
| mIgM probe | TGACTGACTGTCCATGCAGCAACACC |  |
| NFC1 Fw | GTGGAGATTGTGGAGAAAAGC | FJ594437 |
| NFC1 Rev | GACTTCTCCTGCATAGTTGGG |  |
| NFC1 probe | TCTGCCAGTGTGATACCAAGCGG |  |
